# Characterization of Cytokines and Proliferation Marker Ki67 in Cleft Affected Lip Tissue

**DOI:** 10.3390/medicina55090518

**Published:** 2019-08-22

**Authors:** Mara Pilmane, Elga Sidhoma, Ilze Akota, Dzintra Kazoka

**Affiliations:** 1 Institute of Anatomy and Anthropology, Riga Stradins University, Kronvalda Boulevard 9, LV-1010 Riga, Latvia; 2 Institute of Stomatology, Riga Stradins University, Dzirciema Street 20, LV-1007 Riga, Latvia

**Keywords:** cytokines, proliferation marker, lip, children, cleft

## Abstract

*Backgroundandobjectives:* Cleft lip palate takes the second place among all anomalies. The complex appearance of cytokines and proliferation markers has still not been clarified despite their possible crucial role in cleft tissue. Therefore, the aim of work was the detection of appearance of pro- and anti-inflammatory cytokines and proliferation marker Ki67, and their inter-correlations in cleft affected lip (CAL). *Materials* *and* *Methods:* The lip material was obtained from 16 children aged before primary dentition during plastic surgery. Control was obtained from 7 non-CAL oral tissue. Tissues were stained for IL-1, IL-4, IL-6, IL-8, IL-10 and Ki67 immunohistochemically. Non-parametric statistic, Mann–Whitney and Spearman’s coefficient were used. *Results:* All cytokines positive cells were observed more into the epithelium. Statistically significant difference was seen between epithelial IL-1, IL-10, IL-8 and Ki67 positive cells and IL-10-, IL-4-containing connective tissue cells in comparison to the control. Strong positive correlation was detected in CAL epithelium between IL-10 and IL-8, IL-10 and IL-4, IL-10 and IL-1, IL-1 and IL-8, IL-1 and IL-4, IL-4 and IL-8, IL-8 and Ki67, IL-10 and Ki67, but moderate—in connective tissue between IL-1 and IL-10, IL-1 and IL-4. *Conclusions:* The CAL epithelium is the main source for the interleukins. Rich similar expression of IL-1 and IL-10 suggests the balance between pro-and anti-inflammatory tissue response on basis of dysregulated tissue homeostasis (increase of IL-8). The correlations between the different ILs-1, -4, -8, -10 in CAL epithelium seem to indicate the self-protection compensatory mechanism for intensification of local inflammatory-immune response without involvement of IL-6. The correlations between Ki67 and cytokines indicate the involvement of IL-8 and IL-10 in stimulation of cellular proliferation. IL-4 and IL-10 expression from CAL connective tissue simultaneously to IL-1, IL-4 and IL-10 inter-correlations there suggests the intensification of local immune response regulated probably by main pro-inflammatory cytokine—IL-1.

## 1. Introduction

Cleft lip palate (CLP) is one of the most common congenital malformations with estimated incidence at 1 in 600–1000 births worldwide [1,2]. From all anomalies, it occupies the stable second position with slight variations regarding the race, geographic region, environmental and some other probably unknown factors of influence for CLP development. Significance of this disorder is mentioned even in the Global Burden of Disease (GBD) project, which provides longitudinal analysis of the global burden of diseases by measuring the all-cause mortality, years of life lost, the years of life lived with disability, and disability-adjusted life years [3].

Morphopathogenesis of CLP is not completely understood and it is the reason for the ongoing interest of scientists in different developmental and postnatal aspects of clefts. Inflammation and inflammatory cytokines belong to one such aspect. Therefore, children with CLP are known to show greater oral tissue inflammation despite the same persistence of microorganisms than the children without cleft [4]. Relevant studies have indicated the inflammation of tissue as one of the main tissue modulators in the cleft affected regions due to the dysregulated chronic character of inflammation that disturbs the common tissue remodelation in normal wound healing [5]. However, there has been only very limited research done regarding the detection of main indicators of inflammatory process—pro- and anti-inflammatory cytokines into the cleft affected tissue. Some interleukins (ILs) like IL-7 and IL-10 are described as the main immune response regulators in the hard tissue of cleft patients [6], but during the research on the human fibroblasts obtained from CLP the inhibitory effect of IL-6 on the collagen and glycosaminoglycan level was detected [7].

Despite the still scarce studies on the whole cytokine class in CLP affected tissue, interest in the significance of some interleukins: IL-1, -4, -6, -7, -8, and -10 in common body tissue aspect and especially in oral tissue has increased in the last decade. Generally, orofacial tissue develops from craniofacial mesenchyme interacting with immune cells. In return, immune cells attenuate their function by secreting inflammatory cytokines such as interleukin IL-1, mainly IL-1β [8]. Epidermal keratinocytes are also a rich source of ILs, mainly IL-1α, which is released at the time of tissue damage [9]. IL-1α is active as a zymogen and, upon secretion from epidermal keratinocytes, can play key roles in the early inflammatory phase of the wound-healing response [10]. In addition, IL-1α mediates the reciprocal crosstalk between cells within the epidermis and dermis that promotes the secretion of stem cell proliferation factors [11,12]. IL-1 can also be considered among the most common biomarkers that give precise results and can be used as an indicator of periodontal disease progression [13]. Commonly, IL-1 induces expression of macrophage inflammatory protein-1α in polymorphonuclear leukocytes and human gingival epithelial cells and its elevated levels have been reported in inflammatory bone diseases including periodontitis [14].

Interleukin-4 (IL-4) is a pleiotropic cytokine secreted by activated T lymphocytes, mast cells, eosinophils, and basophils. It is the key player in humoral and adaptive immunity by regulating the function of immune cells, such as lymphocytes and macrophages [15,16]. IL-4 is a cytokine that monitors the immuno-inflammatory response associated with severe inflammation [17]. The presence of IL-4-producing cells is significantly higher in periodontal lesions compared to gingival tissues [18]. IL-4 is a common finding in human skin [19]. Finally, also a pro-inflammatory effect of IL-4 in vascular tissues, especially in endothelial cells has been described [20].

IL-6 is mainly regarded as a pro-inflammatory cytokine; meanwhile also anti-inflammatory activities are noted [21]. Pro-inflammatory cytokines are multipotent, critical for cellular homeostasis and play a vital regulatory role in the inflammation process. The overproduction of cytokines can be the root for harmful inflammatory reactions. The upregulation of pro-inflammatory cytokines contributes to enhanced monocyte adhesiveness and infiltration into the skin during the pathogenesis of various inflammatory skin diseases, such as atopic dermatitis [22]. Various cell types including lymphocytes, fibroblasts, endothelial cells and macrophages produce and secrete IL-6 [23]. Keratinocytes also produce a variety of cytokines including IL-6 and tumor necrosis factor-alpha (TNFα). The disparity in cytokine levels can promote the onset of inflammatory diseases [24]. Additionally, IL-6 induces a variety of responses from many cell types. Primary effects include B-cell differentiation and the stimulation of acute phase proteins [25].

IL-8 is a major neutrophil chemotactic factor involved in the acute inflammation [26]. Generally, IL-8 deploys its function in conjunction with other cytokines and chemokines resulting in chemoattraction of leukocytes to the inflammatory sites, recruitment and activation of neutrophils to phagocytosis and bacterial clearance. Furthermore, IL-8 possesses chemoattractant activity on basophils and T cells; additionally, it has a potent pro-angiogenic action. Therefore, IL-8 is crucially involved in several inflammatory diseases [27,28]. Interestingly, inflammation in the oral mucosa might lead to changes in the DNA methylation status of the IL-8 gene in epithelial oral cells [29]. Together with the idea that IL-8 is a significant player in the oral tissue common homeostasis, it is also supposed to explore a notable role in pathological conditions of the tissues [30].

IL-10 is essential for regulating macrophage and neutrophil infiltration as well as cytokine production during the inflammatory response of cutaneous wound healing [31]. IL-10 is an immunoregulatory cytokine with potent anti-inflammatory properties. It can repress the synthesis of major inflammatory cytokines including TNFα, IL-1, IL-6 and IL-12 as well as chemokines [32]. IL-10 may play a remarkable role in wound healing due to the regulation of pro-inflammatory cytokines. Overexpression of IL-10 in adult murine wounds reduces collagen deposition and improves wound healing [33]. Finally, IL-10 is thought to be involved in the regulation of type I collagen synthesis and degradation [34]. As mentioned earlier, all the above-mentioned ILs may modulate the CLP affected tissues and they have been researched only solitarily in the case of this anomaly.

The idea about the impact of high craniofacial tissue proliferation potential indicates the interest in the Ki67 protein and its possible connection to the cytokine-mediated tissue remodelation. The Ki67 protein is well characterized on the molecular level. It is associated with all phases of cell cycle except G0, Ki67 is located in the cortex of the nucleus and is known to be present in all proliferating cells. Therefore, it has been used as a surrogate marker to rate the proliferation index particularly in the diagnosis for carcinogenesis [35]. Interestingly, there are indications regarding slightly different phenotype of oral and lip epithelium where Ki67 also may play a significant role [36]. The common functional significance of Ki67 still remains unclear both in healthy and in cleft affected tissue condition. There are indications, however, that Ki67 protein expression is an absolute requirement for progression through the cell-division cycle and it means that it is absolutely necessary for the proliferation. Despite the fact that nothing is known about this protein and different ILs correlations [37] especially in case of clefts.

Based on the importance of cytokine functions in morphopathogenesis of clefts, absence of their correlative search with proliferation markers in CLP affected tissue, the aim of our work was the research of appearance and distribution of pro- and anti-inflammatory cytokines, proliferation marker Ki67 and the detection of inter-correlations between these factors in cleft affected lip (CAL) of children.

## 2. Materials and Methods

### 2.1. Materials Characteristics of Subjects

This study was independently reviewed and approved by the local Ethical Committee of the Riga Stradins University (22.05.2003; 17.01.2013; 5/25.06.2018), Project Nr.5-1/106/2019 of 12.04.2019) as well as a written informed consent was obtained from all parents after the nature of the study had been fully explained. All of the patients and parents gave their informed consent to participate in the study. Tissues were retrieved from the Cleft Lip and Palate Centre of the Institute of Stomatology, but the study was conducted at the Department of Morphology of the Riga Stradins University, Latvia. The lip material from the defect place was obtained from 16 children (10 boys, 6 girls) aged before primary dentition (3 months to 1.6 years; commonly, the primary dentition age continues until a child turns 6 years old) during plastic surgery of bilateral (2) and unilateral clefts (14). Seven control samples were obtained from lip mucosal part during upper labial frenectomy due to the correction of low attached upper lip frenulum, but the tissues were not associated with CLP, inflammation or any other pathology. Age of control children (4 boys and 3 girls) extended from 5 years to 6 years and 6 months (from all controls, the age of the two oldest children slightly exceeded the primary dentition age, but they still were included into the control group due to the unique character of the tissue as border cases).

### 2.2. Immunohistochemical Analysis

Tissues were fixed for a day in a mixture of 2% formaldehyde and 0.2% picric acid in 0.1 M phosphate buffer (pH 7.2). Afterwards they were rinsed in Tyrode buffer (content: NaCl, KCl, CaCl_2_·2H_2_O, MgCl_2_·6H_2_O, NaHCO_3_, NaH_2_PO_4_·H_2_O, glucose) containing 10% saccharose for 12 h and then embedded into the paraffin. Three micrometres thin sections were cut, which were then stained with hematoxylin and eosin for routine morphological evaluation. Biotin-Streptavidin biochemical method was used for immunohistochemistry (IMH) to detect: IL-1 (orb308737, working dilution 1:100, Biorbyt Limited, Cambridge, UK), IL-4 (orb10908, working dilution 1:100, Biorbyt Limited, Cambridge, UK), IL-6 (sc-130326, working dilution 1:100, Santa Cruz Biotechnology Inc., Santa Cruz, CA, USA), IL-8 (orb 39299, working dilution 1:100, Biorbyt Limited, Cambridge, UK), IL-10 (250713, working dilution 1:100, BioSite, Täby, Sweden), Ki67 (1508202A, working dilution 1:100, Sigma-Aldrich, St. Louis, MO, USA), and anti-macrophage inflammatory protein 1 beta (MIP-1ß, ab9675, working dilution 1:100, Abcam, Cambridge, UK).

The slides were analysed by light microscopy using non-parametric evaluation. The results were evaluated by grading the appearance of positively stained cells in the visual field [38]. Structures in the visual field were labelled as follows: 0—no positive structures, 0/+—occasional positive structures, +—few positive structures, +/++—few to moderate number of positive structures, ++—moderate number of positive structures, ++/+++—moderate to numerous positive structures, +++—numerous positive structures, +++/++++—numerous to abundant structures, ++++—abundance of positive structures in the visual field.

For visual illustration, Leica DC 300F digital camera and image processing and analysis software Image Pro Plus (Media Cybernetics, Inc., Rockville, MD, USA) were used.

### 2.3. Statistical Analysis

The data processing was performed with SPSS software, version 22.0 (IBM Company, Chicago, IL, USA). We used Spearman’s rank correlation coefficient, where r = 0–0.2 was assumed as a very weak correlation, r = 0.2–0.4—a weak correlation, r = 0.4–0.6—a moderate correlation, r = 0.6–0.8—a strong correlation and r = 0.8–1.0—a very strong correlation. To analyse control group versus patient data, we used Mann–Whitney U test. The level of significance for all tests was chosen as 5% (*p*-value < 0.05).

## 3. Results

**Figure 1:**
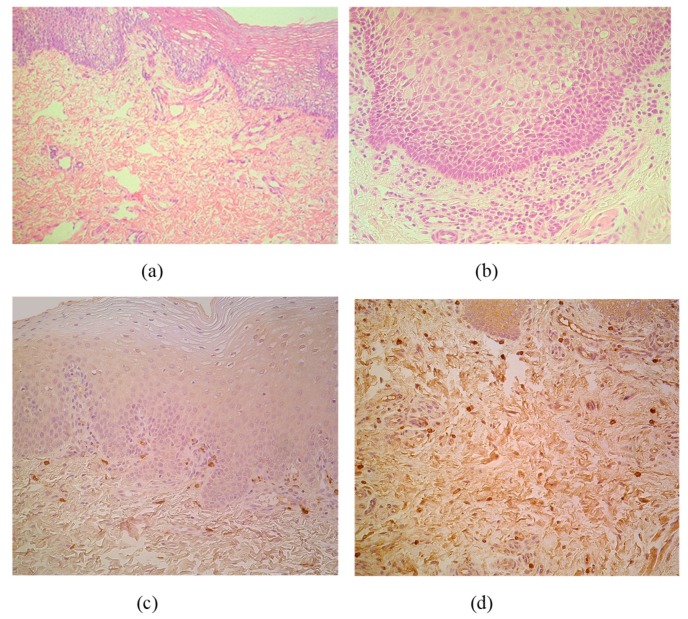
(**a**–**d**) Micrographs of cleft lip affected lip in children. (**a**) Note patchy vacuolization of the lip epithelium (keratosis seborrhoea) in a 3-month-old child. Hematoxylin and eosin, X 200; (**b**) Visible proliferation of the basal epitheliocytes and subepithelial inflammation in a 3-month-old child. Hematoxylin and eosin, X 250. (**c**) MIP-1ß immunoreactive macrophages are located strictly beneath the basal membrane of 5-month-old child. MIP-1ß IMH, X 200; (**d**) Note diffuse distribution of MIP-1ß immunoreactive macrophages in subepithelial connective tissue of 4-month-old child. MIP-1ß IMH, X 200.

The CAL material contained stratified squamous epithelium and submucosal connective tissue in all cases. Routine staining discovered patchy vacuolization of the lip epithelial cells (keratosis seborrhoea), patchy proliferation of the basal cells and patchy subepithelial inflammation with appearance of the sclerosis in small arteries (Figure 1a,b). MIP-1ß marked appearance of pro-inflammatory macrophages or directly beneath the basal membrane or diffusely distributed in subepithelium (Figure 1c,d).

**Figure 2:**
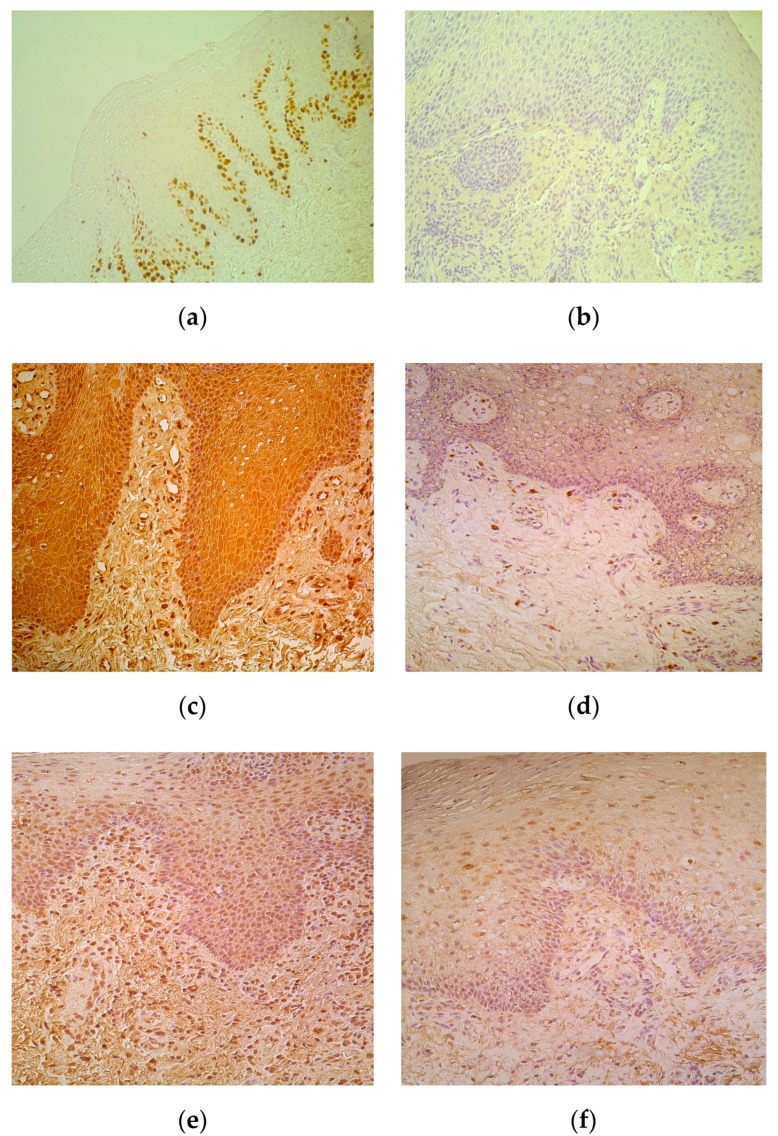
(**a**–**f**) Immunohistochemical micrographs in cleft affected lip of children and in control subjects. (**a**) Note moderate number of basal and suprabasal Ki67 positive cells in epithelium of a 5-month-old cleft affected child. Ki67 IMH, X 200; (**b**) The lip epithelium lacks Ki67 positive cells in control lip of a 5-year-old child. Ki67 IMH, X 200; (**c**) Note numerous number of IL-1-containing epithelial and connective tissue cells of 3-month-old cleft affected child. IL-1 IMH, X 200; (**d**) Moderate number of IL-1 positive connective tissue cells placed in the subepithelium of 6-year-old control child. IL-1 IMH, X 200; (**e**) Moderate number of IL-4 immunoreactive epitheliocytes and connective tissue cells in subepithelium of 4-month-old cleft affected child. IL-4 IMH, X200. (**f**) Note moderate number of IL-4 epithelial cells and only few weakly stained connective tissue cells of 6-year- old control subject. IL-4 IMH, X 200.

Proliferation marker Ki67 marked moderate number of epithelial cells in lip epithelium with five cases when only occasional positive epitheliocytes were observed (Table 1, Figure 2a). No Ki67 positive cells were seen in the control tissue (Figure 2b).

Positive cells of all cytokines were observed more in the epithelium. Moderate to numerous IL-1-containing CAL epitheliocytes prevailed in comparison to few such cells in control (Table 1, Figure 2c), while there was no difference between the numbers of factor positive connective tissue cells, mainly fibroblasts and macrophages (Figure 2d) of patients and controls. IL-4 positive cells slightly varied (from moderate to numerous) in patients’ epithelium, but generally did not differ from the indices in control tissue. Interestingly, patients possessed moderate number of IL-4 immunoreactive connective tissue cells, while controls demonstrated only few of them in the subepithelium (Table 1, Figure 2e,f).

**Figure 3:**
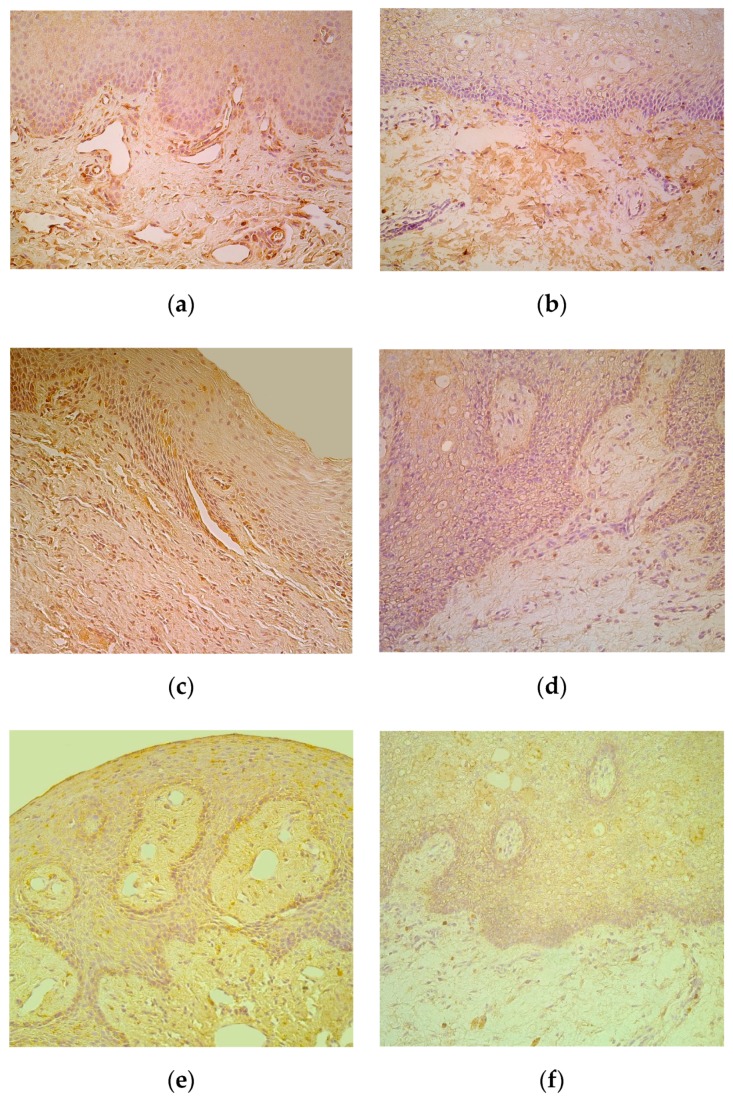
(**a**–**f**) Immunohistochemical micrographs of cleft affected lip in children and in control subjects. (**a**) Note moderate number of IL-6 immunoreactive cells in connective tissue of 1-year-1-month-old cleft affected child. IL-6 IMH, X 200; (**b**) Moderate number of IL-6 positive connective tissue cells in 6-year-old control lip. IL-6 IMH, X 200; (**c**) Note moderate number of IL-8 immunoreactive cells located in the epithelium and connective tissue of 4-month-old cleft affected child. IL-8 IMH, X 250; (**d**) Note moderate number of positive for IL-8 connective tissue cells in 5-year-old control subject. IL-8 IMH, X 200; (**e**) Note moderate number of IL-10 immunoreactive epitheliocytes and connective tissue cells in 3-month-old cleft affected child. IL-10 IMH, X200; (**f**) Five-year-old control subject displaying only a few IL-10 positive connective tissue cells. IL-10 IMH, X 200.

Numerous IL-6-containing epithelial and connective tissue cells in patients notably exceeded the few to moderate number of factor positive cells in control locations (Table 1, Figure 3a,b). IL-8 positive epithelial cells varied in patients with mostly rare appearance, but demonstrated similar appearance of mainly moderate immunoreactive connective tissue cells in patients and controls (Table 1, Figure 3c,d). Moderate to numerous IL-10 positive epithelial cells were detected in patients, while the controls demonstrated only few factor positive epitheliocytes. In addition, connective tissue of patients possessed notably more (++) IL-10-containing cells in comparison to the controls (+) (Table 1, Figure 3e,f).

A statistically significant difference was seen between numbers of epithelial IL-1-, IL-8-, IL-10- and Ki67-containing cells as well as IL-10, IL-4 positive connective tissue cells in comparison to the control (Table 2).

A strong positive correlation was detected in CLP epithelium between IL-10 and IL-8, IL-10 and IL-4, IL-10 and IL-1, IL-1 and IL-8, IL-1 and IL-4, IL-4 and IL-8, IL-8 and Ki67, IL-10 and Ki67, but a moderate correlation was detected in connective tissue between IL-1 and IL-10, IL-1 and IL-4 (Table 3). No correlations were found between factors in controls.

## 4. Discussion

The lip and oral epithelium generally separates the host from the environment and presents as the first line of defense against pathogens, exogenous substances and mechanical stress [39]. From all interleukins researched in our patients, the IL-1, IL-8 and IL-10 were the most expressed ones presenting particularly in the epithelium simultaneously with proliferation marker Ki67. Therefore, we suggest the specific expression/role of these cytokines being the dominant in the cleft affected epithelial barrier, which remarkably stimulates the proliferation of cells. This is proved also by the other scientists who have indicated the role of epithelium as a structure regulating the innate and adaptive immune responses against foreign substances and microorganisms [40]. However, in the case of an oral bacterial infection, mainly the expression of IL-1, IL-6 and also TNFα has been demonstrated to be the most prominent in gingival epithelium up to now [39].

Cleft affected lip tissue shows unequal regeneration as it is regularly affected by mechanical and also bacterial influence, at least in untreated condition. Interestingly, IL-1 is the main pro-inflammatory cytokine, but its expression has been regarded as necessary for healing; however, its importance has also been implicated in delayed wound repair. All these effects take place in CAL. We can agree to the point that there still is no direct evidence on IL-1 central role in the healing [41] and thus its capacity is mainly associated with the persistence of the inflammation. Furthermore, the elevated level of IL-1 supposedly modulates the tissue remodeling up to epithelial proliferation and cellular apical migration, loss of cell attachment, and even destruction of hard tissue in ongoing cytokine expression [42]. IL-8, on the other hand, is known to regulate oral tissue homeostasis [30] and its increase seems to be based on bacterial/viral and mechanical dysregulation of epithelium integrity. Additionally, elevation of cytokines such as IL-1 (as well as TNFα) can also essentially increase the expression of this cytokine [43].

IL-10 was the third most common interleukin in the CAL epithelium in our patients. Initially it was assumed that IL-10 is only produced by immune cells, further studies revealed that IL-10 is produced by non-immune cells as well, including keratinocytes, epithelial cells and tumor cells for instance [32]. As the patchy inflammation was detected in subepithelium in CAL, we speculate of common compensatory inflammation-suppressing expression of IL-10 from epithelial and connective tissue cells in long term persisting cleft raised inflammation circumstances. The main target cells for IL-10 include monocytes and macrophages. IL-10 suppresses the release of pro-inflammatory cytokines and therefore inhibits the secretion of TNF-α and IL-6. IL-10 also suppresses functions of antigen presentation by blocking Major histocompatibility complex (MHC) class II expression and co-stimulatory molecules such as CD80 and CD86, but exalts the phagocytosis of cells. Finally, IL-10 inhibits the release of pro-inflammatory mediators by neutrophils and the secretion of chemokines to attract neutrophils, which is an equally important function at an acute inflammation stage [44].

We detected also strong and moderate correlations between the appearances of different interleukins in the cleft affected epithelium that were not seen in the control. We suppose that this interesting finding indicates the common self-protection compensatory mechanism in the CAL epithelium, which serves as a balance between the regulatory tissue factors in seriously damaged tissue.

Interesting questions are raised due to the elevated Ki67 appearance, which clearly indicates the presence of proliferating cells. We speculate on the triggered influence of some interleukins on the Ki67 expression in CAL and to those belong IL-8 and IL-10 due to their strong and moderate inter-correlations in the epithelium. Our suggestion is indirectly proved also by other authors describing the possible non-inhibitory functions of IL-10. Furthermore, IL-10 can promote the proliferation, differentiation and MHC expression of B cells. IL-10 brings into play its biological effect by binding to its receptor, which is composed of two subunits, IL-10R1 and IL-10R2 [32]. Although the fact that Ki67 shows a high proliferative activity of human dental stem cells in chronic pulpitis during prolonged inflammation and transition to the chronic stage [45] is not new itself. Contrary data have also been obtained in a study where mice were exposed to all-trans retinoic acid to induce cleft palate formation and Ki67 expression was found to be downregulated in mouse embryonic palate mesenchymal cells compared with untreated control mice [46]. Therefore, we suppose that besides the cytokine stimulating effect on Ki67 expression the effects of some additional substances may change the cellular proliferation activity in a different way.

An interesting fact is an absence of Ki67 marked epithelial cells in our control lip. The abnormal Ki67 immunoreactivity pattern was described in metaplastic columnar epithelia of the oesophagus, where positive staining for proliferating cells was observed only in the surface epithelium and superficial third of the foveolar pit [47]. It means that some specific appearance of Ki67 may exist for some distinct type of epithelium. This is supported by the research on cervical epithelium where complete absence or just minimal Ki67 expression was observed in the normal cervical epithelium [48]. Additionally, biomechanical factors, like a masticatory pressure might influence the phenotype of oral mucosal epithelium and change the proliferation ability in epithelium [49]. As the control lip material investigated by us enrols only seven cases we can just speculate that probably the upper lip frenulum tissues belong to some specific region in proliferation ability/intensity aspect what should be more clarified in future studies with more cases.

Connective tissue of subepithelium in CAL demonstrated significant dominance of IL-4- and IL-10-containing inflammatory and non-inflammatory cells, while very strong and moderate inter-correlations were observed between IL-1 and IL-4, IL-1 and IL-10, and IL-4 and IL-10 positive cells. Therefore, we suppose that from all cytokines in case of cleft triggered inflammation, the IL-1, -4 and -10 are those, which are the result of the local tissue response. This suggestion of ours is supported also by the research on the cytokines in periodontal tissue. Hence, IL-10 serves for the control of periodontal disease progression and the large diversity of its expression pattern in periodontal tissues may be affected by various micro-environment factors. Significantly negative correlation exists between the levels of IL-1 and IL-10 in clinically healthy periodontal tissues of the patients and dynamic relationship between pro- and anti-inflammatory cytokines may be crucial in periodontal homeostasis and pathogenesis [50]. Despite the separate remarks on the effects of IL-4 on oral tissue cells—still not well enough examined—and the increased IL-4 level in healthy periodontal tissue [51,52], the data about the elevated expression of IL-4 and IL-10 secreted by different cells is quite common in current scientific literature. Additionally, gingival atypical fibroblasts in cases of Chédiak–Higashi syndrome significantly intensify the expression of IL-4 and IL-10 along with other cytokines. Scientists indicate the possibly dysregulated immunoinflammatory response in such fibroblasts that may partly lead to the development of periodontitis [53]. Additionally, in periodontitis granulation tissue Th2 lymphocytes secrete cytokines eliciting humoral type immunity—i.e., IL-4 and IL-10. Simultaneously IL-4 could be also secreted by Th0 cells. Th2 cells are less effective in promoting microbial killing compared to Th1 cells as IL-4 and IL-10, products of these cells, downplay these activities by inhibiting the secretion of IFN-γ. As a result, Th2-dominated response reduces cellular immunity in the presence of certain bacterial infections, particularly if host cells are invaded and may be associated with failed control of the infection [54]. Finally, in periapical granulations around the site of inflammation the significant presence of IL-4 and IL-10 shows the active involvement of the inflammatory cells in the downregulation of the inflammatory response and modulation of further disease activity [55].

This function could be related to the IL-4 role in common leukocyte survival under pathological (also physiological) conditions [56], and tissue repair and homeostasis through the so-called “alternative” macrophage activation, i.e., a pathway of macrophage activation that differs from the classical pro-inflammatory pathway [57]. On the other hand, hypoxia seems to be a factor, which selectively can influence the specific cytokines like IL-10, as human gingiva-derived mesenchymal stem cells when exposed to hypoxia show increase in expression of IL-10, in this manner contributing to the promotion of immunomodulation and optimizing tissue regeneration [58].

An interesting issue is related to the ontogenetic changes of cytokines, which were not an aim of our study of the cleft affected tissues and mainly are studied in periodontal tissues. As follows, in vivo research it has been revealed that during the cellular aging the levels of IL-4 decreased as well as increased levels of IL-1, IL-6 and slight increase for IL-8 has been shown in the periodontal cells, suggesting a link between various age-associated diseases and chronic inflammatory state. However, the age-related decrease on proliferation of periodontal ligament cells has a major role in periodontal homeostasis [59], and it is probably not a topical question in cleft affected children periodontium and/or lip.

## 5. Conclusions

Commonly, the cleft affected epithelium is the main source for the interleukin expression. Rich similar expression of pro-inflammatory cytokine IL-1 and anti-inflammatory cytokine IL-10 suggests the balance between pro-and anti-inflammatory tissue response on the basis of possibly dysregulated tissue homeostasis (increase of IL-8).

The detected strong and moderate correlations between the appearances of different interleukins (ILs-1, -4, -8, -10, but not IL-6) in the cleft affected epithelium seem to indicate the common self-protection compensatory mechanism for intensification of local inflammatory-immune response without involvement of all pro-inflammatory cytokines (IL-6). The correlations between Ki67 and cytokines indicate the involvement of IL-8 and IL-10 in stimulation of cellular proliferation.

IL-4 and IL-10 expression from CAL connective tissue cells, mainly macrophages and fibroblasts simultaneously to IL-1, IL-4 and IL-10 inter-correlations there suggests the intensification of local tissue immune response regulated probably by main pro-inflammatory cytokine—IL-1.

## Figures and Tables

**Table 1:** Relative number of cytokines and proliferation marker Ki67 positive structures in the cleft affected lip epithelium and subepithelial connective tissue.

Factors/Subjects	Ki67	IL-1		IL-4		IL-6		IL-8		IL-10	
	E	E	CT	E	CT	E	CT	E	CT	E	CT
1.	++/+++	+/++	+	+++	++	+++	++	++/+++	+++	+++	+++
2.	0/+	+++	++	+++	++	++++	++	+++	+++	+++	+++
3.	0/+	+	++	++	++	+/++	0	0	0	+/++	+
4.	0	0	+/++	+	+	0	++	++	+/++	+	+
5.	++/+++	++	+++	+	+++	++	+++	0	++	++	+++
6.	++	+++	++	++	+++	+++	+++	++	++	+++	++
7.	++	+++	++	+++	+++	+++	+++	+++	+++	+++	++
8.	++/+++	+++	++	+++	+++	+++	+++	+/++	++	+++	++
9.	+++	+++	++	+++	+++	+++	+++	0	++	+++	++
10.	++/+++	++++	++	+++	+++	+++	+++	0	+	++++	+++
11.	0/+	+/++	++	+	+	+/++	++	0/+	++/+++	++	++
12.	++	+++	++	+++	+	+++	+++	++	++	+++	++
13.	0/+	++	++	0	+	+/++	++	0	+	++	++
14.	++	+++	+++	+++	+++	++/+++	+++	0/+	+++	++/++	+++
15.	++	++	++	++	++	++	++	+/++	+++	++	++
16.	+/++	+++	++	+++	++	++	+++	+/++	+++	++	+/++
**Subjects common**	++ *	++/+++ *	**++**	**Var ++/+++**	++ *	**+++**	**+++**	Var +-+++ *	**++/+++**	++/+++ *	++ *
**Control**	**0**	**+**	**++**	**++**	**+**	**+/++**	**++**	**+/++**	**++**	**+**	**+**

Abbreviations: E—epithelium; CT—connective tissue; Ki67—proliferation marker; IL-1—interleukin 1; IL-4—interleukin 4; IL-6—interleukin 6; IL-8—interleukin 8; IL-10—interleukin 10; 0/+—occasional positive structures, +—few positive structures, +/++—few to moderate number of positive structures, ++—moderate number of positive structures, ++/+++—moderate to numerous positive structures, +++—numerous positive cells, +++/++++—numerous to abundant structures, ++++—abundance of positive structures in the visual field; Var—variable appearance of positive structures; * statistically significant difference of the relative number in structures between the subjects and controls.

**Table 2:** Mann–Whitney U test revealing statistically significant differences in factors of different locations between the patients and controls.

Detected Factor	Mann–Whitney U	Z-Score	*p*-Value
IL-1 in epithelium	14.5	2.06431	0.01394
IL-8 in epithelium	15.5	2.35907	0.01828
IL-10 in epithelium	20.5	2.33854	0.01928
Ki67 in epithelium	10.5	3.00669	0.00262
IL-4 in connective tissue	16	2.32221	0.02034
IL-10 in connective tissue	6	3.30736	0.00094

Abbreviations: Ki67—proliferation marker; IL-1—interleukin 1; IL-4—interleukin 4; IL-8—interleukin 8; IL-10—interleukin 10.

**Table 3:** Spearman’s rank correlation coefficient revealed correlations between the relative numbers of different tissue factors in CLP affected epithelium and connective tissue.

Factor 1	Factor 2	R	*p*-Value
Very strong positive correlation			
IL-10 in epithelium	IL-8 in epithelium	0.92791	0
Strong positive correlation			
IL-10 in epithelium	IL-4 in epithelium	0.77857	0.00038
IL-10 in epithelium	IL-1 in epithelium	0.72389	0.00152
IL-1 in epithelium	IL-8 in epithelium	0.77684	0.0004
IL-1 in epithelium	IL-4 in epithelium	0.68943	0.00313
IL-4 in epithelium	IL-8 in epithelium	0.72045	0.00164
Ki67 in epithelium	IL-8 in epithelium	0.64175	0.00736
IL-10 in connective tissue	IL-4 in connective tissue	0.65084	0.00632
Moderate positive correlation			
Ki67 in epithelium	IL-10 in epithelium	0.56539	0.02246
IL-1 in connective tissue	IL-4 in connective tissue	0.55068	0.02707
IL-10 in connective tissue	IL-1 in connective tissue	0.51299	0.04214

Abbreviations: Ki67—proliferation marker; IL-1—interleukin 1; IL-4—interleukin 4; IL-10—interleukin 10.
